# Negative studies are helpful to compute the specificity of diagnostic tests: measuring *Trypanosoma cruzi* seroprevalence in Guanajuato, Mexico

**DOI:** 10.1186/s13104-015-1612-z

**Published:** 2015-10-28

**Authors:** Nicolás Padilla-Raygoza, Rubí Gamboa-León, Maria Jesus Ramirez-Sierra, Eric Dumonteil, Pierre Buekens, Ma Laura Ruiz-Paloalto, Rosalina Diaz-Guerrero

**Affiliations:** División de Ciencias de la Salud e Ingenierías, Departamento de Enfermería y Obstetricia, Campus Celaya Salvatierra Universidad de Guanajuato, México Mutualismo 303, CP38060 Celaya, Gto Mexico; Coordinacion Académica Región Huasteca Sur, Universidad Autónoma de San Luis Potosí 5 km Carr, Tamazunchale-San Martin, C.P. 79960 Tamazunchale, SLP Mexico; Laboratorio de Parasitologia, Centro de Investigaciones Regionales “Dr. Hideyo Noguchi”, Universidad Autónoma de Yucatán, Ave. Itzaes #490 × 59, 97000 Merida, Yucatán Mexico; School of Public Health and Tropical Medicine, Tulane University, 1440 Canal St., Ste. 2430, 70112 New Orleans, LA USA; Departamento de Enfermería Clínica, División de Ciencias de la Salud e Ingenierías, Campus Celaya Salvatierra, Universidad de Guanajuato, Celaya, Gto Mexico; División de Ciencias de la Salud e Ingenierías, Departamento de Enfermería y Obstetricia, Campus Celaya Salvatierra, Mutualismo 303, CP38060 Celaya, Gto Mexico

**Keywords:** *Trypanosoma cruzi*, Antibodies, Specificity

## Abstract

**Background:**

Publishing negative seroprevalence studies not only helps to have more accurate seroprevalence estimates but also allows calculating the specificity of the diagnostic tests used. We performed a population-based *Trypanosoma cruzi* seroprevalence survey in a community in central Mexico.

**Results:**

We surveyed 204 women and children and collected blood by finger prick. We performed rapid tests (Stat-Pak, Chembio, Inc., Medford, New York) and recombinant Chagas ELISA tests v3.0 (Wiener, Rosario, Argentina). All rapid tests and all ELISA tests were negative.

**Conclusion:**

The rapid test had 100 % of specificity compared to the ELISA.

## Results

Results of negative seroprevalence studies are often not published. Finding no infected individual in a community might be disappointing to the infectious diseases researcher, which might induce a strong publication bias. Yet, publishing such negative studies not only helps to have more accurate seroprevalence estimates but also allows calculating the specificity of the diagnostic tests used. Specificity is defined as the proportion of non-infected individuals correctly identified as negative by the test [1]. The results of the test under evaluation need to be compared to a gold standard: at least two tests need to be performed on each sample to calculate specificity.

Chagas’ disease, or American trypanosomiasis, is caused by the protozoan parasite *Trypanosoma cruzi* and is a major cause of morbidity and mortality in endemic countries in Latin America. It is estimated that 6–7 million people are infected in Latin America [2]. Two positive serological tests are needed to confirm chronic Chagas’ disease. There is some uncertainty about *T. cruzi* seroprevalence in Mexico [3–6]. In 2008, we performed a population-based household *T. cruzi* seroprevalence study among women of reproductive age and their children in Estancia del Llano, Guanajuato, Mexico, where we had identified two seropositive pregnant women in a previous study [7]. In 2005, Estancia del Llano had a population of 1859 inhabitants, 203 of whom were children under 5 years and 530 of whom were women of reproductive age, including 17 pregnant women [8]. We used Epi Info 3.3.2 (Centers for Disease Control, Atlanta, GA) to calculate that a sample of 185 women of reproductive age and children would allow us to measure a seroprevalence of 0.5 % with a possible high result of 2 %, with a 99 % confidence interval and a 1.5 design factor taking into account the cluster sampling. We surveyed 177 homes from 13 blocks in the community. We contacted 204 people living within the selected households; 42 of them were under 5 years, 157 were non-pregnant women of childbearing age (12–49 years), and five were pregnant women. Data were collected on forms designed specifically for the study. Labels with de-identified study numbers were pasted on data collection forms and blood samples.

After obtaining informed consent, blood was collected by finger prick. Stat-Pak (Chembio, Inc., Medford, New York) rapid tests were performed and filter paper was impregnated with capillary blood for further study. The filter papers collected each day were stored in the General Hospital Celaya clinical laboratory and kept frozen at −20 °C until shipment to the laboratory of parasitology, Centro de Investigaciones Regionales (CIR) “Dr. Hideyo Noguchi” at the Autonomous University of Yucatán, where recombinant ELISA Chagas Wiener (Rosario, Argentina) v3.0 was used according to the manufacturer’s instructions. Samples were assayed in duplicate on two different ELISA plates, with each plate including one blank (without sample), two positive controls (human serum with antibodies anti-*T. cruzi*), and three negative controls (human serum without antibodies anti-*T. cruzi*) from the manufacturer (Table 1). The corrected Optical Densities (ODs) were obtained with OD sample less OD blank. The results of the two plates were validated because the optical densities were <0.150 OD for at least two corrected negative controls and ≥0.600 for the average corrected positive controls (Table 1). The cut-off for each ELISA plate was the average OD of the corrected negative control +0.3, as defined by the manufacturer’s instructions, and the indeterminate zones were determined as specified by the manufacturer as well.

**Table 1:** Results of the ELISA plates for 204 blood samples

	ELISA plate #1	ELISA plate #2
Blank	0.035	0.036
Corrected negative control 1	0.011	0.002
Corrected negative control 2	−0.003	0.008
Corrected negative control 3	0.003	−0.002
Average positive controls	1.000	1.000
Cut off	0.303	0.302
Cut off ± 10 % (indeterminate)	Upper limit = 0.334 Lower limit = 0.273	Upper limit = 0.333 Lower limit = 0.272
Corrected sample results (n = 204)	−0.014 to 0.026	−0.003 to 0.022

All 204 rapid tests were negative. Using the ELISA as gold standard, we calculated a 100 % specificity for the Stat-Pak rapid test. The specificity of 100 % rapid test study is consistent with the specificity reported by Sosa et al. for the same rapid test compared to the same gold standard: 99.4 % in Argentina, 98.6 % in Bolivia, 97.5 % in Honduras, and 99.6 % in Mexico [7]. We concluded that the Stat-Pak is a highly specific rapid test for use in population-based surveys in Mexico and is useful to rule out Chagas’ disease infection in different geographic areas. The results suggest that the prevalence of Chagas’ disease is low in Guanajuato, Mexico.

The protocol was reviewed and approved by Research and Bioethics Committee of the School of Nursing and Obstetrics of Celaya, University of Guanajuato, and by the IRB of the School of Public Health and Tropical Medicine of Tulane University in New Orleans, USA.
